# Mechanisms by Which B Cells and Regulatory T Cells Influence Development of Murine Organ-Specific Autoimmune Diseases

**DOI:** 10.3390/jcm6020013

**Published:** 2017-01-26

**Authors:** Jason S. Ellis, Helen Braley-Mullen

**Affiliations:** 1 Department of Surgery, University of Missouri, Columbia, MO 65212, USA; ellisj@health.missouri.edu; 2 Department of Molecular Microbiology & Immunology, University of Missouri, Columbia, MO 65212, USA; 3 Department of Medicine, University of Missouri, Columbia, MO 65212, USA

**Keywords:** B cells, autoimmunity, regulatory T cells, effector T cells, APC

## Abstract

Experiments with B cell-deficient (B−/−) mice indicate that a number of autoimmune diseases require B cells in addition to T cells for their development. Using B−/− Non-obese diabetic (NOD) and NOD.H-2h4 mice, we demonstrated that development of spontaneous autoimmune thyroiditis (SAT), Sjogren’s syndrome and diabetes do not develop in B−/− mice, whereas all three diseases develop in B cell-positive wild-type (WT) mice. B cells are required early in life, since reconstitution of adult mice with B cells or autoantibodies did not restore their ability to develop disease. B cells function as important antigen presenting cells (APC) to initiate activation of autoreactive CD4+ effector T cells. If B cells are absent or greatly reduced in number, other APC will present the antigen, such that Treg are preferentially activated and effector T cells are not activated. In these situations, B−/− or B cell-depleted mice develop the autoimmune disease when T regulatory cells (Treg) are transiently depleted. This review focuses on how B cells influence Treg activation and function, and briefly considers factors that influence the effectiveness of B cell depletion for treatment of autoimmune diseases.

## 1. Introduction

Autoimmune diseases develop as a result of loss of tolerance of self-reactive CD4+ T cells. Development of a number of organ-specific autoimmune diseases in mice and humans, e.g., type I diabetes, multiple sclerosis, autoimmune thyroid diseases, rheumatoid arthritis, collagen induced arthritis, systemic lupus erythematosus, and Sjogren’s syndrome require the contribution of B cells. The importance of B cells for development of autoimmune diseases is illustrated by the fact that B cell deficient (B−/−) mice are resistant to autoimmune diseases that develop in their B cell-positive counterparts spontaneously or after immunization with self-antigen [1,2,3,4,5,6,7,8,9] and by numerous reports indicating that B cell depleting agents inhibit development of several autoimmune diseases in both mice and humans [4,7,10,11,12,13,14]. B cells contribute to autoimmune diseases by producing autoantibodies [15,16], releasing proinflammatory cytokines such as Interleukin (IL)-6, and suppressive cytokines such as IL-10 and IL-35 [12,17,18].They also function as antigen presenting cells (APCs) for activation of autoreactive T cells [2,19,20,21,22]. In some autoimmune diseases, best illustrated by experimental allergic encephalomyelitis (EAE), B cell depletion can promote development of more severe disease through the activity of regulatory B cells (Breg) [23,24,25,26]. However, in some EAE models and in humans with multiple sclerosis, B cell depletion can inhibit or slow the progression of disease [27,28,29,30], indicating that B cells have both positive and negative effects on autoimmune diseases. Here, we will review available evidence demonstrating important interactions between autoreactive effector T cells (Teff), regulatory T cells (Treg) and B cells in development of organ-specific autoimmune diseases in several murine models of autoimmune disease. The role of regulatory B cells has been addressed in recent reviews [31,32], and will be addressed very briefly here.

## 2. B Cells Are Important APC for Activation of Autoreactive T Cells

As discussed above, a requirement for B cells for development of a particular autoimmune disease has frequently been addressed either by generating mice that completely lack B cells (B−/− mice) or by depleting B cells in adult mice by administration of antibodies directed against CD20 or other surface molecules on B cells. Several studies, including those from our laboratory, indicate that the ability of B cells to function as APC for activation of autoreactive T cells is one of the most important roles for B cells in development of autoimmune diseases. B cells have several characteristics that make them particularly effective and efficient APCs. B cells capture and bind specific antigen through their surface Immunoglobulin (Ig) receptors prior to internalization and processing. Therefore, if antigen (self-antigen in the case of autoimmune diseases) is limiting, antigen uptake by the B cell receptor effectively results in a greater amount of antigen being taken up and processed by autoantigen-specific B cells compared to other APC such as dendritic cells and macrophages, since the latter cells do not have surface Ig receptors with the ability to specifically bind antigen [12,27,33]. This property can also be important for perpetuation of some autoimmune diseases. For example, autoantigen-specific B cells in the target organ can efficiently present limited amounts of antigen to lower the threshold for activation of Teff, thus promoting chronic T cell activation and inflammation [16,28,33]. Experiments with transgenic mice engineered to have B cells that express surface Ig but do not secrete antibody also indicated that B cells have an important role as APC that is not dependent on their ability to secrete autoantibody [3,34,35]. The ability of B cells to promote autoimmune disease is dependent on their expression of the major histocompatibility complex (MHC) class II molecules required for antigen presentation to CD4+ T cells [19,36,37], expression of MHC class I molecules for activation of CD8+ T cells [22] and expression of costimulatory molecules required for effective T cell activation [21]. In some studies, B cells with specificity for a particular autoantigen were shown to be critical for development of autoimmune disease [2,20,38,39].

## 3. Increased Function of Tregs in the Absence of B Cells Provides One Explanation for the Requirement for B Cells for Development of Autoimmune Diseases

Studies from our laboratory as well as others have shown that B−/− mice, normally resistant to several autoimmune diseases, develop the disease when Tregs are depleted [5,34,40,41,42]. We used a mouse model (Non-obese diabetic (NOD).H-2h4) that spontaneously develops autoimmune thyroiditis (SAT) when given NaI in their drinking water. B−/− NOD.H-2h4 mice do not develop SAT, and the B cell requirement cannot be replaced by antithyroglobulin (Tg) autoantibodies [1]. Importantly, B cells are required primarily in the first 4–6 weeks after birth since mice given B cells as adults do not develop SAT [1]. Because B cells are rejected by host CD8+ T cells when transferred to adult mice [1,2], B−/− mice were sublethally irradiated prior to B cell transfer. B cells were transferred to 8–9 weeks old mice and full reconstitution of the splenic B cell compartment was confirmed at the end of the experiment 8 weeks later (Table IV in [1]). Moreover, T cells from B−/− mice could function as effector cells for SAT when they developed from bone marrow precursors in the presence of B cells [1]. The results suggest that B cells are required for the early activation of CD4+ effector T cells, functioning as important APC or to amplify T cell responses through production of particular cytokines. Because the defect in B−/− mice cannot be corrected by providing B cells to adults, we hypothesized that if the initial presentation of autoantigen to naïve CD4+ T cells occurs in the absence of B cells, they are unable to induce SAT when B cells are provided to adults [1]. One explanation for these results is that the initial encounter of naïve T cells with autoantigen did not result in expansion, so when B cells were provided to adults at 8 weeks of age, autoantigen-specific T cells were less activated compared to T cells in 8 week old mice that developed from birth in the presence of B cells. Our results did not directly address this possibility. We also postulated that the inability of T cells from B−/− mice to function as Teff for SAT might be due to Treg activity [34]. Indeed, multiple autoimmune diseases develop spontaneously when Treg are absent, as in scurfy mice [43,44], or reduced, as in day 3 thymectomized mice [45]. SAT in NOD.H-2h4 mice is inhibited by the activity of Treg [46,47,48].

To determine if Treg activity could explain the resistance of B−/− mice to SAT, mice were given three weekly injections of the anti-CD25 antibody PC61 beginning at 10 days of age. At 8 weeks, they were given NaI water and thyroids were removed 8 weeks later. The results were clearcut; the B−/− mice given anti-CD25 developed SAT with an incidence and severity comparable to that of wild-type (WT) NOD.H-2h4 mice [34]. In contrast to WT mice, anti-CD25 treated B−/− mice had no detectable anti-Tg autoantibodies indicating that the presence of autoantibodies does not contribute to SAT severity scores. All WT mice with SAT produce anti-Tg autoantibodies and autoantibody levels typically correlate with SAT severity scores. In addition, clusters of T and B cells that form germinal center-like tertiary lymphoid organs, common in WT mice with SAT [49,50] are clearly not present in the thyroids or spleens of anti-CD25 treated B−/− mice.

We subsequently carried out similar studies in NOD mice. WT NOD mice develop several organ-specific autoimmune diseases, including diabetes, autoimmune thyroid disease and Sjogren’s syndrome, whereas B−/− NOD mice are resistant to these diseases [5]. A similar course of administration of anti-CD25 to B−/− NOD mice resulted in development of all three diseases in B−/− NOD mice [5]. We and others showed that Treg activity was largely responsible for suppression of disease when WT mice were depleted of B cells using anti-CD20 [14,24,41,42,51] or other treatments that deplete mature B cells [40]. Results in humans also indicate that Tregs can increase after B cell depletion with rituximab [24]. Taken together, these results are all consistent with the hypothesis that Treg can be preferentially activated in comparison to Teff if T cells initially encounter their cognate (self) antigen in the absence of B cells, whereas if the initial encounter occurs when B cells are present, Teff are effectively activated. In a rotavirus-induced model of biliary atresia, B−/− mice were resistant to disease and had increased numbers of Treg, but depletion of Treg did not result in development of the disease [52], indicating that other mechanisms can contribute to disease resistance in B−/− mice in some models.

## 4. Treg Numbers and Function Differ in the Presence or Absence of B Cells

The results described above, although compelling, are not observed in all models. For example, several studies asked if the presence or absence of B cells influences total numbers of peripheral Tregs, and results were inconsistent. In our studies, WT and B−/− NOD.H-2h4 mice had similar absolute numbers of peripheral Foxp3+CD4+ T cells [34]. However, when B cells were depleted by anti-CD20, Treg numbers were approximately 2-fold higher than in mice with B cells [41]. The percentage of CD4+ T cells expressed as a percentage of total lymphocytes is greater when B cells are depleted because of the absence of a major cell population (B cells). Because the percentage of the CD4+ T cell population that expressed Foxp3 did not change after B cell depletion, the absolute numbers of Treg were higher when B cells were reduced or absent [41]. Several other reports are consistent with our studies, indicating that Foxp3+ Treg numbers increase after B cell depletion by anti-CD20 and Treg were largely responsible for suppression of the autoimmune disease [14,24,40,42,51,52,53]. Other reports indicate that Treg activation occurs normally in the presence or absence of B cells [54]. B cells can also promote expansion of Treg [25,55,56,57,58], including Treg that do not express Foxp3 [59,60], and several studies showed that B−/− mice had reduced numbers of Treg compared to their B cell-positive counterparts [24,25,61,62]. Reduced Tregs in B−/− mice have most often been reported in models where B cells play a regulatory role in suppressing the autoimmune disease, e.g., models of EAE in which B cell depletion promotes development of more severe disease [24,61] and in some tumor models in which B cells inhibit tumor growth [63]. It is difficult to reconcile these disparate results, although the particular autoimmune model being studied, or the mouse strain or inflammatory environment may determine whether Treg numbers are expanded, reduced or unaffected by B cell depletion. B cells can impact autoimmune diseases by multiple mechanisms, some of which are Treg-independent, as discussed above.

## 5. Treg in Mice Lacking B Cells Differ Functionally from Those in B Cell-Positive Mice

**Figure 1:**
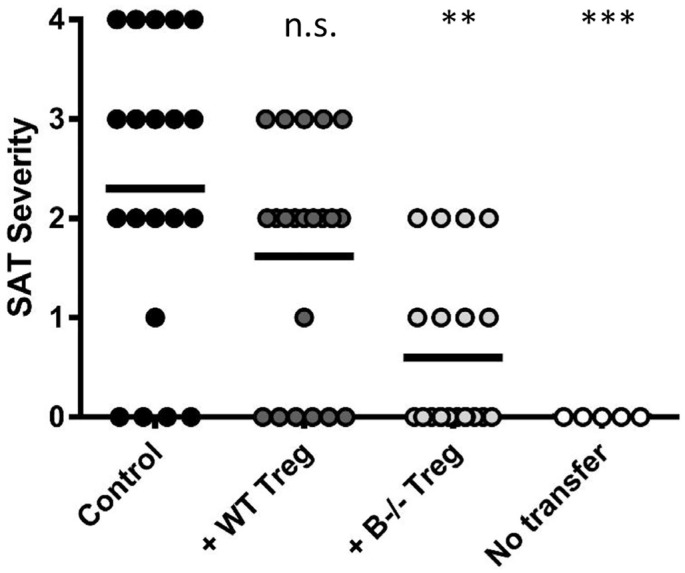
T regulatory cells (Treg) from B−/− mice are more effective at suppressing spontaneous autoimmune thyroiditis (SAT) than Treg from wild-type (WT) mice. T cells from CD28−/−B−/− Non-obese diabetic (NOD).H-2h4 mice were cultured with or without Treg from WT or B−/− NOD.H-2h4 mice. TCR−/− NOD.H-2h4 recipients were given 5 × 10^6^ cultured T cells, and were given NaI in their drinking water. The no transfer group did not receive T cells. Thyroids were removed 8 weeks later. ** *p* < 0.01; *** *p* < 0.001, n.s., not significant. Results are the mean SAT severity scores from individual recipient mice. See [63] for additional details.

Our results indicating that Treg depletion results in autoimmune disease in B−/− mice that are normally resistant to those diseases [5,34,41] suggests that Treg in B−/− mice could have superior suppressor function compared to Treg in B cell-positive mice. It is also possible that activated Teff in B cell-positive mice are relatively resistant to suppression by Treg or that Treg become unstable in an inflammatory environment [64]. To determine if Treg in WT and B−/− NOD.H-2h4 mice differ in their ability to suppress activation of Teff that induce SAT in NOD.H-2h4 mice, we used a protocol in which Treg from WT and B−/− mice interacted in vitro with a single population of Teff and non-B APCs before transfer to mice that had B cells but no T cells [65]. The results indicated that Treg from B−/− mice were more effective than the same number of Treg from WT mice in suppressing activation of Teff for SAT (Figure 1). Both Treg populations proliferated and survived to a comparable extent in the recipient mice indicating that Treg populations differ functionally in WT and B−/− mice [65]. Our results are consistent with those reported by another group in a mouse model of experimental arthritis [42,53], but functional differences between Treg in WT and B−/− mice were not observed in models of EAE [54] or diabetes [14].

Our experiments showed that Treg in WT and B−/− mice, in addition to differing in function, had significant differences in cell surface expression of several molecules, including glucocorticoid induced tumor necrosis factor related protein (GITR), Tumor Necrosis Factor Receptor II (TNFRII) and CD27 [65]. Importantly, if T cells from B−/− mice developed from bone marrow precursors in the presence of bone marrow from B cell-positive mice, Treg had the phenotype of WT Treg and not Treg from B−/− mice [65]. Unfortunately, attempts to correlate the phenotypic differences with differences in function were not successful. In the mouse model of experimental arthritis where Treg from B−/− mice had increased function compared to Treg from WT mice, production of Interferon (IFN)-γ by B cells was reported to be responsible for the inhibition of Treg function and development of more severe arthritis [53]. These results are of particular interest because IFN-γ is a proinflammatory cytokine, and other proinflammatory cytokines such as IL-6 [66,67], IL-2 [66], granulocyte macrophage colony stimulating factor (GM-CSF) [30] and TNF-α [68], all of which can be produced by B cells, can interfere with Treg function and could contribute to increased Teff activation when B cells are present. B cell production of IFN-γ or other proinflammatory cytokines could contribute to the ability of B cells to function as effective APC for activation of autoreactive Teff [66]. B cells also express molecules such as GITR-L which can block Treg expansion or function in some models [69,70,71,72]. However, GITR-L expressed on B cells was also reported to maintain Tregs at a level sufficient to inhibit EAE [25], and GITR can be a marker for functional Treg [73]. Therefore, signaling through GITR can have different outcomes depending on the environment and/or activation state of Treg and Teff [71].

In most autoimmune disease models, T cells in B−/− mice will usually be in a less inflammatory environment than they are in B cell-positive mice, and the inflammatory environment may be a major factor in determining the differential functions of Treg in WT vs. B−/− mice. When the inflammatory environment is high, Breg can become activated in an attempt to downregulate the inflammation, e.g., by producing anti-inflammatory cytokines such as IL-10 and IL-35 [74,75,76]. Cytokines produced by Breg inhibit activation or expansion of Teff, and can promote expansion of Treg [31,77,78,79]. Therefore, Breg play an important role in dampening autoimmunity in several different models, most notably in EAE where they have been extensively studied [26,31,77,79,80]. Overall, these results suggest that B cells and/or specific molecules produced or expressed by B cells can both inhibit and promote Treg function in some autoimmune disease models. Further studies are needed to determine the specific cytokines or cell surface molecules that are most important in this regard.

## 6. Transient Depletion of Treg Is Sufficient to Result in Autoimmune Disease in B−/− Mice Because Tregs That Repopulate Following Depletion Have Reduced Function

**Figure 2:**
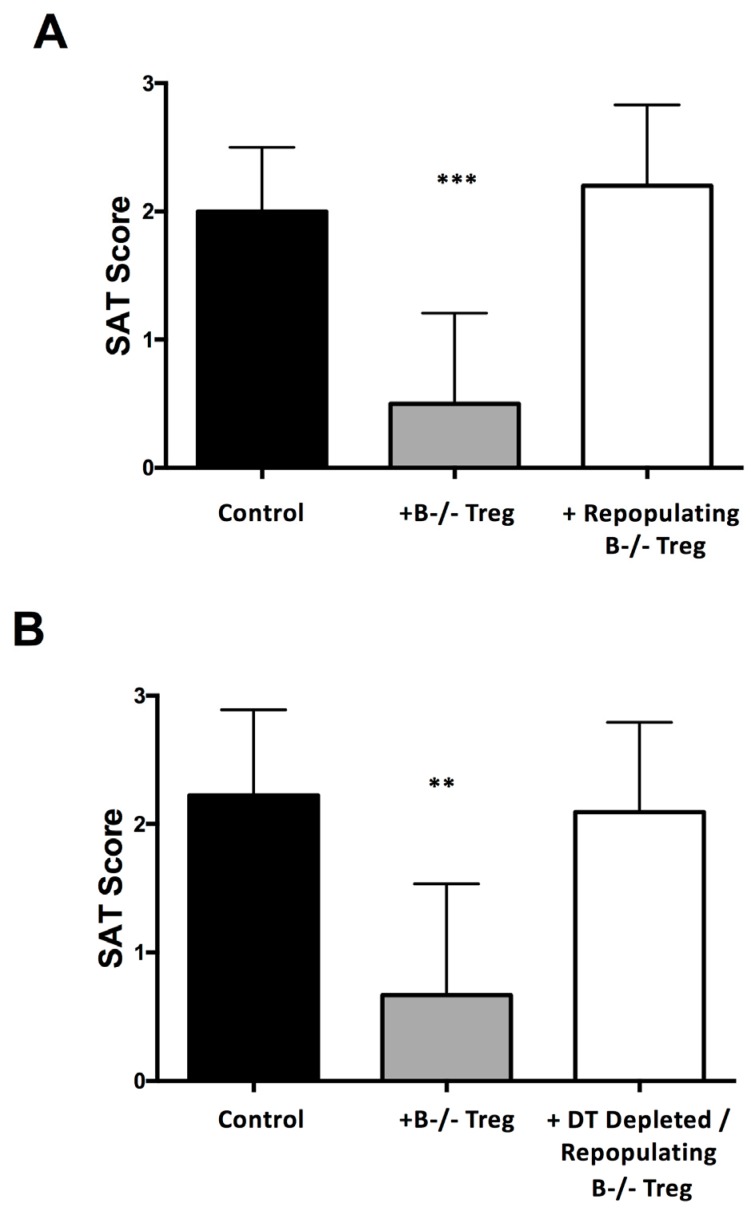
Repopulating Treg have reduced suppressive function compared to endogenous Treg. Treg were depleted by injection of anti-CD25 (Foxp3-GFP.NOD.H-2h4 mice) or diphtheria toxin (DT) (Foxp3-DTR NOD.H-2h4 mice. Treg were allowed to repopulate the spleen, and repopulating or control (endogenous) Treg were cultured with naïve T cells from CD28−/−B−/− NOD.H-2h4 mice. Groups of TCR−/− NOD.H-2h4 recipient mice were given T cells cultured with or without Treg, and all mice were given NaI water for 8 weeks. (**A**): *n* = 11 mice/group; (**B**): *n* = 9 mice/group. ** *p* < 0.01; *** *p* < 0.001. Results are mean SAT severity scores from groups of 9–11 recipients ± SEM. Additional details are in [63].

The fact that Treg depletion results in development of autoimmune diseases in B−/− mice that are normally resistant to those diseases is perhaps not unexpected given that mice lacking Treg due to absence of Foxp3+ T cells spontaneously develop several organ-specific autoimmune diseases and die at a young age [43,81]. In the studies described above, where Treg depletion leads to autoimmune disease in B−/− mice that normally do not develop the disease, the situation is different. First, administration of anti-CD25 generally results in reduction of CD25+CD4+ T cells for less than 2 weeks [5,34,41,65]. In some studies, anti-CD25 reduced both CD25+ and Foxp3+ cells suggesting that Tregs are actually depleted [5,14,42,65], whereas another study showed that anti-CD25 did not deplete Foxp3+ cells but functionally inactivated them [82]. In any event, Treg depletion by anti-CD25 is likely to be less complete than in Foxp3-negative scurfy mice, and Treg depletion or inactivation is transient, since anti-CD25 is administered for a relatively short time. In our experiments, mice were given 3 weekly injections of anti-CD25 beginning at 3–4 weeks of age, and most Treg repopulated the peripheral lymphoid organs 7–14 days later Similar results were obtained using Foxp3diphtheria toxin receptor (DTR) mice, in which Treg are clearly depleted and not inactivated, the duration of Treg depletion was shorter than with anti-CD25 [65]. Since the autoimmune diseases in our model and others develop over several months, relatively normal numbers of Treg are present during most of the time disease is developing, yet they fail to inhibit disease development. These results suggest that Treg that repopulate the host after anti-CD25 depletion may have reduced function compared to endogenous Treg that are present prior to depletion, or that Teff that become activated when Treg are absent or low are relatively resistant to suppression by Treg [83]. Our studies provided clear evidence that Treg that repopulate after administration of anti-CD25 or diphtheria toxin (DT) in Foxp3-DTR mice had reduced ability to suppress activation of Teff compared to endogenous Treg (Figure 2) [65]. In another model, Treg lost their ability to suppress development of autoimmune gastritis after anti-CD25 depletion and repopulation [83,84,85], suggesting that reduced Treg function could be relatively common when Tregs are transiently depleted, In addition to having reduced suppressive function, Treg repopulating the spleens of anti-CD25 or Foxp3-DTR mice differed phenotypically from endogenous Treg in B−/− mice, having surface markers like those of the less functional Treg in untreated WT mice [65]. Importantly, transient Treg depletion differs from the global and permanent Treg depletion in scurfy mice that develop multiorgan inflammation, because mice in which Treg are depleted transiently develop inflammation only in the organs that are normally affected by autoimmunity in that genetic background.

If Treg that repopulate lymphoid organs after depletion by anti-CD25 typically have reduced suppressive function, this would have major implications for situations where Treg inhibit beneficial responses, notably in tumor models. In fact, transient Treg depletion is sufficient to promote long-lasting tumor immunity in many different tumor models, suggesting that repopulating Treg may generally be less capable of suppressing Teff [86,87,88,89,90]. One study showed that Treg depletion in humans resulted in loss of suppressive function and reprogramming of repopulating Treg to Foxp3+Treg that produced IFN-γ [91]. Others have also described Foxp3+Treg that produce IFN-γ, demonstrating the plasticity of these cells and their ability to become Th1-like in certain scenarios [92,93,94] Another group showed that a unique Teff population with characteristics of T follicular helper cells, expanded in the absence of Treg, leading to loss of B cell anergy and increased autoantibody responses [95,96,97,98]. While none of these studies rule out the possibility that failure of repopulated Treg to suppress anti-tumor responses or autoimmune responses could also be due to a reduced susceptibility of activated Teff to suppression by Treg [83,99], they do indicate that prolonged Treg depletion is not necessary to promote development of immune responses dampened by endogenous Treg. The results also suggest that the reduced function of repopulating Treg could be due to phenotypic changes [65] and/or to reprogramming of Treg to produce proinflammatory cytokines that result in reduced suppressive function [91]. Further studies are needed to understand why repopulating Treg tend to have reduced function compared to endogenous Treg.

## 7. Multiple Factors Influence the Effectiveness of B Cell Depletion by Anti-CD20

The importance of B cells in autoimmune diseases has been well documented by the effectiveness of B cell depletion therapy for treating autoimmune diseases in humans and in mouse models. Among the most widely used B cell-depleting agents are those that target the CD20 molecule expressed on essentially all mature B cells [100,101]. Rituximab, directed against CD20 on human B cells, has been used to treat autoimmune diseases in humans [102,103,104] and antibody specific for mouse CD20 has been used to prevent or treat autoimmune diseases in many mouse models [3,13,14,25,41,42,61,105,106]. The effectiveness of anti-CD20 for suppression of autoimmune disease varies in different mouse models and human diseases, and several factors that influence the effectiveness of B cell depletion are briefly reviewed here. For example, the isotype of the anti-CD20 antibody (IgG1 vs. IgG2a) influences the effectiveness of B cell depletion by anti-CD20, with the IgG2a isotype being more effective [13,107]. One reason for the greater efficacy of IgG2a anti-CD20 for B cell depletion is due to its ability to at least partially deplete marginal zone (MZ) B cells that are almost completely resistant to depletion by IgG1 anti-CD20 [41,107]. Rituximab also does not effectively deplete MZ B cells in humans [24]. MZ B cells are increased and may have an important function in some murine models of autoimmunity [108,109,110,111]. Although their pathogenic role is unknown, the resistance of MZ B cells to depletion by anti-CD20 could explain the ineffectiveness of B cell depletion therapy in some cases. The effectiveness of MZ B cell depletion can also influence whether B cells have to be continuously depleted or whether transient depletion of B cells is sufficient to suppress some autoimmune diseases [41]. MZ B cells usually develop later than other B cell subsets in mice [112]. We showed that if anti-CD20 was administered before most MZ B cells developed, B cell depletion was more complete, and adult mice did not develop SAT even if B cell depletion was not maintained [41]. Alternatively, continued B cell depletion may have been unnecessary because repopulating B cells might function as Breg to suppress disease [62]. It is also possible that B cell depletion in very young mice that will spontaneously develop an autoimmune disease is more effective because activation of potential Teff is prevented before they would typically become activated in a normal untreated mouse. In this regard, B cell depletion in NOD mice effectively prevents diabetes development when anti-CD20 is administered early in life, before autoreactive B cells enter pancreatic islets, whereas depletion after B cells enter the pancreas or after diabetes develops is ineffective, due in part to loss of CD20 expression on islet-infiltrating B cells [14].

Breg reportedly have low CD20 expression, and are relatively resistant to depletion by anti-CD20 [113], so depletion of CD20+ B cells could enrich for Breg. This could be beneficial if Breg are suppressing an autoimmune disease. Plasma cells also express little or no surface CD20 and are generally resistant to depletion by anti-CD20 [24,114]. If plasma cells and/or autoantibody produced by plasma cells are playing a pathogenic role in a particular disease [114], anti-CD20 could lead to enrichment of plasma cells and worsen the disease.

Follicular (FO) B cells comprise the major subset of circulating B cells and the major subset of splenic and lymph node B cells. FO B cells express high levels of CD20 and are usually effectively depleted by anti-CD20 [14,24,39,53,105,107,115]. Because they circulate, FO B cells are the major B cell subset that infiltrates target organs such as the thyroid and pancreas in mouse models of autoimmune disease, and are the predominant B cell subset in spleen and lymph node. We and others have shown that when FO B cells enter the thyroid or pancreas, they lose CD20 expression and are no longer susceptible to depletion by anti-CD20 unless the re-enter the circulation [14,39,105]. In our studies, anti-CD20 effectively depleted the thyroid-infiltrating B cells, because they cells re-entered the circulation and re-expressed CD20 and could be depleted. If B cells were prevented from re-entering the circulation, they were not depleted by anti-CD20 [105]. Pancreatic B cells that lost CD20 expression were not depleted by anti-CD20 [14], perhaps because they do not recirculate like thyroid-infiltrating B cells. Non-circulating B cells in inflamed joints, bone marrow and solid tissues in humans may also be less susceptible to Rituximab compared to circulating B cells [115,116,117]. The mouse strain and activation status of B cells are also important factors that influence the effectiveness of B cell depletion therapy [106,115]. Because it is important to target a wide range of B cell subsets and B cells in tissues for effective treatment of autoimmune diseases, several recent studies have used combinations of anti-CD20 and other reagents such as B cell activating factor (BAFF) blockade with greater success than seen with either treatment alone [118,119].

## 8. Summary/Concluding Remarks

**Figure 3:**
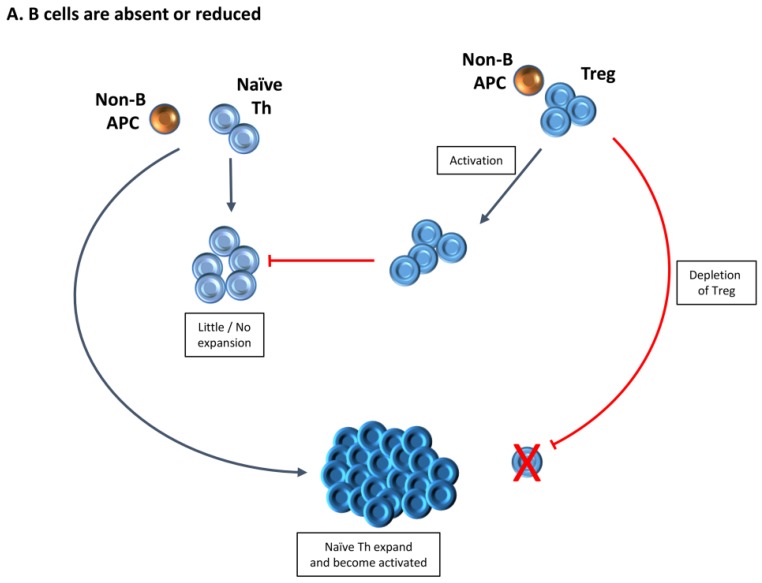

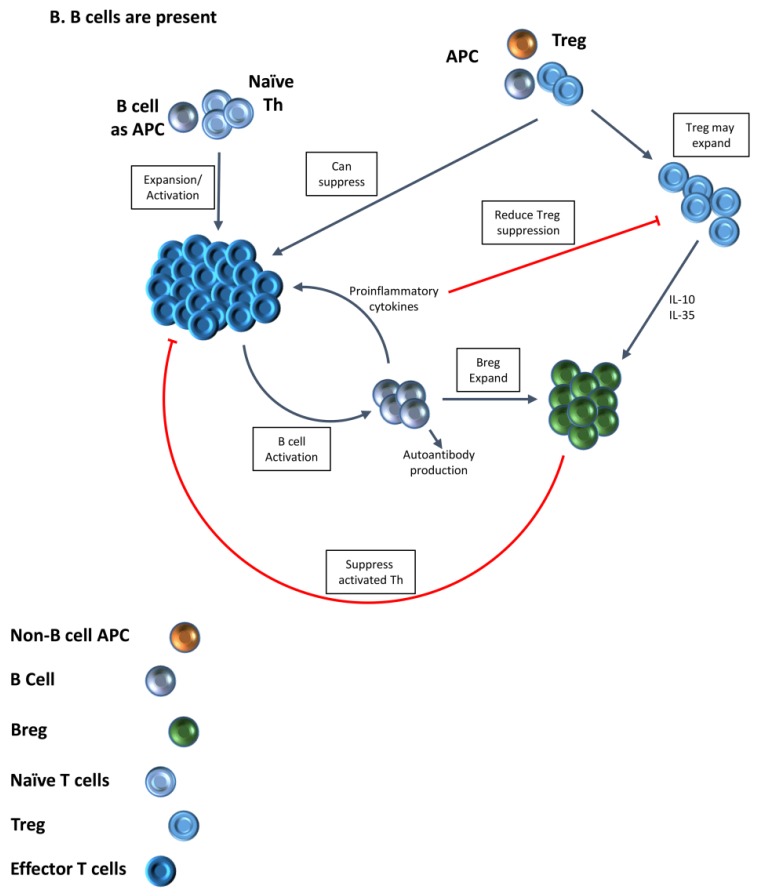
Interactions between Treg, B cells and effector T cells (Teff) in development of autoimmune diseases. (**A**) When B cells are absent or reduced in number (e.g., after anti-CD20 treatment), B cells are not available to present autoantigen. Presentation of autoantigen by other antigen-presenting cells (APC) results in little expansion or activation of naïve T helper (Th) cells and they do become Teff. However, Treg may expand and they are activated, and autoimmune disease does not develop because Treg activity is dominant. When Treg are depleted, presentation of autoantigen by non B cells leads to activation and expansion of naïve Th, they become Teff and autoimmunity develops; (**B**) B cells are present and able to function as APC. Naïve Th expand and become activated. Presentation of antigen to Treg may also lead to their activation, but Th activation is dominant. Suppression by Treg is insufficient to prevent autoimmune disease. Over time, Treg can produce Interleukin (IL)-10 and IL-35 leading to expansion of B regulatory cells (Breg) which suppress activated Th and effector B cells. Expanded Th produce proinflammatory cytokines and interact with B cells to promote their activation and production of autoantibody. Proinflammatory cytokines produced by B cells can also interfere with Treg function and/or activation, rendering suppression by Treg less effective. The dominant outcome is autoimmune disease.

In this review, we focused primarily on murine models of autoimmune disease that indicate the importance of B cells and the multiple mechanisms by which they can regulate autoimmune diseases. In particular, we highlight the importance of Treg in controlling development of autoimmunity both when B cells are present and when they are absent or reduced, as in B−/− mice or after B cell depletion therapy. Two important messages that have derived from our studies and those of others are the fact that Treg that repopulate a host after depletion by anti-CD25 or DT have reduced function compared to endogenous Treg. Also, CD20-positive B cells can lose CD20 expression after migrating to a target organ, and if those B cells do not re-enter the circulation, they will not be depleted by anti-CD20. Figure 3 summarizes some of the possible interactions between B cells, Treg and Teff discussed in this review.
